# Trajectories and predictors of high-occurrence pain flares in ambulatory cancer patients on opioids

**DOI:** 10.1093/jncics/pkae003

**Published:** 2024-01-24

**Authors:** Salimah H Meghani, Ryan Quinn, Andrew Robinson, Jesse Chittams, Neha Vapiwala, Mary Naylor, Martin Cheatle, George J Knafl

**Affiliations:** Department of Biobehavioral Health Sciences; NewCourtland Center for Transitions and Health, University of Pennsylvania, Philadelphia, PA, USA; Abramson Cancer Center, University of Pennsylvania, Philadelphia, PA, USA; Leonard Davis Institute of Health Economics, University of Pennsylvania, Philadelphia, PA, USA; Department of Biobehavioral Health Sciences; NewCourtland Center for Transitions and Health, University of Pennsylvania, Philadelphia, PA, USA; Department of Biobehavioral Health Sciences; NewCourtland Center for Transitions and Health, University of Pennsylvania, Philadelphia, PA, USA; Department of Biobehavioral Health Sciences; NewCourtland Center for Transitions and Health, University of Pennsylvania, Philadelphia, PA, USA; Abramson Cancer Center, University of Pennsylvania, Philadelphia, PA, USA; Department of Biobehavioral Health Sciences; NewCourtland Center for Transitions and Health, University of Pennsylvania, Philadelphia, PA, USA; Leonard Davis Institute of Health Economics, University of Pennsylvania, Philadelphia, PA, USA; Perelman School of Medicine, University of Pennsylvania, Philadelphia, PA, USA; School of Nursing, University of North Carolina at Chapel Hill, Chapel Hill, NC, USA

## Abstract

**Background:**

Pain flares have a substantive impact on the quality of life and well-being of patients with cancer. We identified longitudinal trajectories (clusters) of cancer pain flares in ambulatory patients and sociodemographic and clinical predictors of these trajectories.

**Methods:**

In a prospective cohort study using ecological momentary assessment (mEMA), we collected patient-reported daily pain flare ratings data over 5 months and identified predictors and correlates using validated measures.

**Results:**

The mean age of the sample (N = 270) was 60.9 years (SD = 11.2), 64.8% were female, and 32.6% self-identified as African American. Four pain flare clusters were identified. The “high-occurrence” cluster (23% of patients) experienced 5.5 (SD = 5.47) daily flares, whereas low-moderate clusters (77%) reported 2.4 (SD = 2.74) daily flares (*P* < .000). Those in the high-occurrence cluster reported higher pain scores (*P* = .000), increased pain-related interference (*P* = .000), depressive symptoms (*P* = .023), lower quality of life (*P* = .001), and reduced pain self-efficacy (*P* = .006). Notably, 67.2% of those prescribed opioids as needed (PRN only) were in the high-occurrence pain flare cluster, compared with 27.9% with PRN and around-the-clock opioid prescriptions (*P* = .024). Individual predictors of high-occurrence pain flares were income below $30 000, unemployment, being African American, lower education level, Medicaid insurance, current opioid misuse (COMM), baseline inpatient hospital stay duration, and PRN-only opioid regimen. In the multiple predictor model, lower education level, unemployment, COMM score, extended inpatient duration, and PRN-only opioid regimen remained significant.

**Conclusion:**

In ambulatory patients with cancer, high occurrence of pain flares may be mitigated by attention to opioid prescription factors and addressing social determinants of health needs of underserved patients.

Cancer pain has a profoundly negative impact on patients’ daily lives and function (1,2). Especially devastating are episodic spikes of cancer pain appearing with a greater intensity (3,4). Unfortunately, this recurrent worsening pain is highly prevalent in cancer patients, with 40% to 81% of ambulatory patients experiencing several episodes of intensified pain daily (5-8). This phenomenon is characterized by a rapid onset to peak intensity (within minutes), with sharp or intense pain lasting up to an hour (9).

The phenomenon of acute intensification of cancer pain has been described with many labels in the literature, including breakthrough cancer pain (BTcP) or breakthrough pain (BTP), episodic pain, transient pain, pain exacerbation, intermittent pain, acute-on-chronic pain, spike pain, and pain flares (3,6,10-12). For example, the National Cancer Institute (NCI) defines pain flares interchangeably with BTcP, a definition influenced by the seminal work of Portenoy and Hagen (13). Despite conceptual overlap, as well as similarities in its episodic nature, acute intensification, and rapid onset, BTcP typically occurs against a backdrop of relatively controlled and stable baseline pain and analgesia (3,13), whereas pain flares may also occur in the context of poorly controlled baseline pain or unstable regimen of analgesia (3).

On the basis of accumulated research, poorly controlled cancer pain is common in oncology patients, especially among racial and ethnic minorities and socially vulnerable groups (14-19) who experience both higher background pain levels (16,18,20) and different or inferior cancer pain management strategies (16,18,21,22). Importantly, in the context of an ongoing opioid crisis, the approach to around-the-clock (ATC) analgesic coverage also has changed in recent years. Between 2013 and 2017, opioid prescriptions by oncologists decreased significantly, in line with downward trends observed among non-oncologists (23,24) and partly attributed to a notable 28.5% decline in long-acting (LA) opioid prescriptions used for ATC pain management (23). These changes also align with stricter opioid regulations and changing attitudes among oncologists toward their use (24,25).

In 2016, North America represented the region most dramatically impacted by the opioid crisis, with a rate 3 times higher than the global average (26). In response, a number of strategies were introduced in the United States to reduce opioid prescribing. These included the federal opioid prescription guidelines (27), programs aimed at reducing opioid prescription by clinicians, and state laws restricting opioids (28,29), prescription drug monitoring programs, and health-system-level opioid stewardship programs (30-32)—all aimed at curbing opioid prescribing. Although these strategies successfully reduced rates of opioid prescribing, adverse repercussions of these programs are not fully understood.

The Centers for Disease Control and Prevention’s (CDC’s) 2016 guidelines included cancer patients “outside of active cancer treatments” as part of its scope (27). These guidelines recommended use of nonopioid therapies as first-line treatment for chronic pain patients and encouraged use of short-acting opioids if necessary. However, this distinction overlooked pain trends in cancer survivors (33,34). A longitudinal study on 4903 cancer survivors identified pain as a top-3 symptom affecting quality of life in the first postdiagnosis year. Notably, pain levels were comparable among those who were still receiving active cancer treatments and those who had completed active cancer treatment (33). Although the updated 2022 CDC opioid guidelines excluded cancer patients from its scope altogether (35,36), major changes affecting oncology pain management practices resulting from earlier guidelines appear to be persisting.

Thus, monitoring ongoing trends in clinical treatment of cancer pain is essential to ensure patients’ symptom management needs are met. However, it is not clear how opioid prescription patterns and social determinants of health (SDoH) variables relate to acute pain intensification (pain flares). Among ambulatory patients with cancer, we investigated (1) patient-level pain flare trajectories and unique longitudinal clusters of daily cancer pain flares and (2) if opioid prescription and SDoH factors predicted experience of high-occurrence pain flares over time.

## Methods

### Study design

This study was based on data from a larger, ongoing prospective observational study (1R01NR017853) to examine long-term relationships among patterns of opioid use, patient-reported outcomes, and health-care use in ambulatory patients with cancer. The data for this analysis were collected between November 2019 and November 2022. A repeated-measures survey was administered at 5 timepoints (ie, T1 = baseline; T2 = 1 month; T3 = 2 months; T4 = 3 months, T5 = 5 months), and daily ecological momentary assessments (mEMA) for pain severity, pain flares, and opioid self-management were collected at each timepoint. In addition, relevant clinical variables were extracted from the review of electronic medical records corresponding to the study timepoints. The study was approved by the University of Pennsylvania’s Institutional Review Board (IRB protocol #833009). The study followed recommended STROBE guidelines for reporting of observational studies (37).

### Study participants

Participants were recruited from the outpatient oncology clinics of a large health system in the mid-Atlantic region consisting of 6 hospitals and including an NCI-designated cancer center. Participants were older than 18 years of age, self-identified as African American or White, had a non-skin malignancy, and were prescribed at least 1 extended release or 2 subsequent immediate-release opioid prescriptions in the past 6 months for ongoing pain. Eligible participants provided at least 10 days’ worth of pain flares data using mEMA, which was considered analytically necessary to estimate pain flares’ trajectories over time. Patients were excluded if they were receiving opioids only for treatment of opioid use disorder or procedural pain, residing in a nursing home or receiving hospice care, or had cognitive deficits or medical conditions that interfered with informed consent.

### Study measures

The key study outcome was the patient-reported rating of the number of pain flares on daily mEMA assessment collected using a previously validated question: “How many pain flares or attacks did you have in the past 24 hours?” (38). The terminology “pain flare” was purposefully chosen, as it aligns with the language commonly used by patients as a lay term for BTP and conveys a distinct intensification of preexisting pain. Ratings ranged from 0 to 10, with 10 meaning 10 or more pain flares. The NCI definition of pain flare was used to offer additional context for respondents, that is, “A sudden increase in pain that may occur in patients who already have chronic pain from cancer … Breakthrough pain usually lasts for a short time. During breakthrough pain, the level of pain may be severe but the type of pain and where it is in the body were usually the same as the patient’s chronic pain” (39).

The following validated self-reported measures were administered at survey timepoints to assess for pain severity and function (Brief Pain Inventory Short Form) (40), health-related quality of life (Functional Assessment of Cancer Therapy–General [FACT-G]) (41), depression (Patient Health Questionnaire) (42,43); opioid misuse (Current Opioid Misuse Measure [COMM]) (44), alcoholism (Cut down, Annoyed, Guilty, Eye-opener [CAGE]) (45,46), and use of complementary and alternative modalities to manage pain on the basis of National Center for Complementary and Integrative Health categories (47). Patient-reported data also included age, sex, race, Hispanic ethnicity, marital status, living status, education, household income, employment status, and health insurance type. In addition, the electronic medical record for each patient was reviewed at baseline and at each study timepoint to assess cancer type, stage and metastatic disease, comorbidities, number, types, frequencies, and doses of unique opioid prescriptions.

### Data analyses

#### Participant-level pain flare trajectories

First, to facilitate understanding of pain flare variability across time, participant-level pain flare trajectories were categorized into 6 types: constant, increasing, decreasing, concave, convex, and oscillating. Next, variability was considered by calculating means and half-widths of error bands for each participant’s pain flares over 10 equally spaced times. These 20 vectors, comprising 10 mean estimates and 10 half-width estimates, represented trajectories. Trajectories were matched for participants at proportional study times, allowing for different participation times. The 20 vectors were adaptively clustered into trajectory patterns, using a mixture of multivariate normal distributions for each cluster. Clusters had distinct mean and standard deviation vectors, but shared correlation matrices to simplify the model. Likelihoods and likelihood cross-validation scores were computed for clustering alternatives, considering 1-10 clusters using various methods. Clustering options required cluster sizes to be at least 10% of the sample size to avoid sparse clusters. The selected clustering alternative was the one generating the highest likelihood cross-validation score, determined through analysis (48,49). Subsequent analysis focused on dichotomous indicator of highest pain flare cluster compared with lower pain flare clusters.

#### Pain flare clusters

Proportional odds logistic regression models based on cumulative logits were used to characterize increasing levels of pain flares. Sociodemographic, pain, opioid, and other relevant predictors of pain flares were used to generate individual predictor models for high-occurrence pain flare trajectories. To assess composite predictor effects, the multiple logistic regression model based on all individually significant variables was computed, assessed for multicollinearity using R^2^ values, and then adjusted using backward elimination to identify a unique set of predictors of high-occurrence pain flares. Further, participant characteristics between those in the highest identified pain flare cluster and those in the other low-moderate pain flare clusters were compared using 2-sample *t* tests and χ^2^ tests for continuous and categorical measures, respectively. Statistical analysis was performed using SAS 9.4 for Windows. An alpha level of 0.05 was used for determining statistical significance.

## Results

Of the 379 participants in the parent study at the time of this analysis, 317 provided mEMA data for 1-150 days of study participation. Of these, 270 (82.5%) provided at least 10 pain flare daily measurements (Table 1; Supplementary Figure 1, available online). The final sample of 270 participants was primarily female (64.8%), and one-third of the participants were African American (32.6%). Breast (20%) and lung (12.2%) were the most common types of cancers, with 45.6% participants diagnosed with either stage III or stage IV cancer. Mean age was 60.9 (SD, 11.23), and participants reported mean pain flares of 3.1 (SD, 3.75). Participants provided a range of 10-255 days of daily pain flare data. The majority of participants (n = 266, 98.52%) reported at least one pain flare during the observation period. One-third of participants (N = 91, 33.7%) reported at least 1 pain flare on each day of observation. More than half of the participants were prescribed PRN-only opioids (51.7%), and 39.8% were prescribed both ATC + PRN opioids; the remaining 8.6% were prescribed LA/ATC opioids without PRN coverage (Table 2).

**Table 1.** Baseline characteristics of participants and distribution by pain flare clusters

Baseline characteristic		Total (N = 270)	High pain flares (n = 62)	Low-moderate pain flares (n = 208)	*P*
Age mean (SD)	Years	60.9 (11.23)	60.1 (11.16)	61.1 (11.27)	.557
BHLS score	Mean (SD)	12.2 (3.08)	12.0 (3.11)	12.3 (3.08)	.592
Sex	Male	95 (35.2%)	22 (35.5%)	73 (35.1%)	1.000
	Female	175 (64.8%)	40 (64.5%)	135 (64.9%)	
Race	African American	88 (32.6%)	26 (41.9%)	62 (29.8%)	.089
	White	182 (67.4%)	36 (58.1%)	146 (70.2%)	
Ethnicity	Hispanic or Latino	5 (1.9%)	3 (4.8%)	2 (1.0%)	.081
	Non-Hispanic or non-Latino	265 (98.1%)	59 (95.2%)	206 (99.0%)	
Marital status	Married	147 (54.4%)	26 (41.9%)	121 (58.2%)	.069
	Separated, divorced, widowed	65 (24.1%)	18 (29.0%)	47 (22.6%)	
	Never married	58 (21.5%)	18 (29.0%)	40 (19.2%)	
Education	High school (9-12) or less	109 (40.4%)	32 (51.6%)	77 (37.0%)	.125
	College/Trade (13-16)	119 (44.1%)	23 (37.1%)	96 (46.2%)	
	More than college (>17)	42 (15.6%)	7 (11.3%)	35 (16.8%)	
Household income	<$30 000	83 (30.9%)	30 (48.4%)	53 (25.6%)	.004
	$30 000-$89 999	108 (40.1%)	20 (32.3%)	88 (42.5%)	
	≥$90 000	78 (29.0%)	12 (19.4%)	66 (31.9%)	
Employment status	Employed	57 (21.1%)	8 (12.9%)	49 (23.6%)	.001
	Unemployed, disabled	114 (42.2%)	39 (62.9%)	75 (36.1%)	
	Retired	99 (36.7%)	15 (24.2%)	84 (40.4%)	
Insurance status	Private	103 (38.4%)	16 (25.8%)	87 (42.2%)	.027
	Medicare	126 (47.0%)	32 (51.6%)	94 (45.6%)	
	Medicaid	39 (14.6%)	14 (22.6%)	25 (12.1%)	
Cancer type	Lung	33 (12.2%)	8 (12.9%)	25 (12.0%)	.910
	Breast	54 (20.0%)	12 (19.4%)	42 (20.2%)	
	Colon	13 (4.8%)	4 (6.5%)	9 (4.3%)	
	Prostate	15 (5.6%)	4 (6.5%)	11 (5.3%)	
	Other	155 (57.4%)	34 (54.8%)	121 (58.2%)	
Cancer stage	I/II	82 (30.4%)	15 (24.2%)	67 (32.2%)	.459
	III/IV	123 (45.6%)	32 (51.6%)	91 (43.8%)	

**Table 2.** Pain, opioid, and clinical characteristics by pain flare clusters^a^

Characteristic	Total (N = 270)	High pain flares cluster (n = 62)	Low-moderate pain flares cluster (n = 208)	*P*
Pain-related variables (mean, SD)				
Pain flares	3.1 (3.75)	5.5 (5.47)	2.4 (2.74)	.000
BPI: Severity	4.5 (2.03)	5.5 (2.14)	4.2 (1.89)	.000
BPI Worst Pain	6.2 (2.33)	7.5 (2.08)	5.9 (2.28)	.000
BPI Least Pain	3.0 (2.35)	3.9 (2.88)	2.7 (2.10)	.001
BPI: Interference	4.8 (2.56)	6.2 (2.26)	4.4 (2.49)	.000
BPI: Interference	4.8 (2.56)	6.2 (2.26)	4.4 (2.49)	.000
NPQ Pain to Touch	30.6 (33.57)	44.0 (39.62)	26.6 (30.52)	.000
Opioid management (No. %)				
MME category				
≤25 MME	46 (17.0%)	12 (19.4%)	34 (16.3%)	.125
26-50 MME	71 (26.3%)	22 (35.5%)	49 (23.6%)	
51-90 MME	74 (27.4%)	11 (17.7%)	63 (30.3%)	
90+ MME	79 (29.3%)	17 (27.4%)	62 (29.8%)	
Opioid prescription				
PRN only	139 (51.7%)	41 (67.2%)	98 (47.1%)	.024
Both PRN + LA/ATC	107 (39.8%)	17 (27.9%)	90 (43.3%)	
LA/ATC only	23 (8.6%)	3 (4.9%)	20 (9.6%)	
Opioid risks (mean, SD)				
COMM score	6.6 (5.18)	8.4 (6.18)	6.0 (4.72)	.001
ORT OUD score	1.6 (1.58)	1.7 (1.68)	1.6 (1.55)	.585
CAGE score mean	0.1 (0.49)	0.2 (0.66)	0.1 (0.43)	.477
Alcohol problems (CAGE) (No. %)				
No	260 (96.3%)	59 (95.2%)	201 (96.6%)	.701
Yes	10 (3.7%)	3 (4.8%)	7 (3.4%)	
Pain self-management				
Cannabis use				
No cannabis	173 (64.1%)	39 (62.9%)	134 (64.4%)	.638
Medical	68 (25.2%)	14 (22.6%)	54 (26.0%)	
Recreational	20 (7.4%)	6 (9.7%)	14 (6.7%)	
Medical and recreational	9 (3.3%)	3 (4.8%)	6 (2.9%)	
CBD-alone products				
No	226 (83.7%)	53 (85.5%)	173 (83.2%)	.845
Yes	44 (16.3%)	9 (14.5%)	35 (16.8%)	
Acupuncture				
No	249 (92.6%)	56 (91.8%)	193 (92.8%)	.784
Yes	20 (7.4%)	5 (8.2%)	15 (7.2%)	
Cognitive behavioral therapy				
No	261 (97.4%)	58 (96.7%)	203 (97.6%)	.655
Yes	7 (2.6%)	2 (3.3%)	5 (2.4%)	
Physical therapy				
No	74 (27.4%)	20 (32.3%)	54 (26.0%)	.334
Yes	196 (72.6%)	42 (67.7%)	154 (74.0%)	
PSEQ score	32.2 (14.36)	27.8 (15.21)	33.5 (13.87)	.006
Clinical outcomes				
Quality of Life (FACT-G)	67.5 (17.62)	61.1 (18.50)	69.4 (16.93)	.001
Sleep (PSQI Global Score)	10.6 (3.62)	10.8 (3.45)	10.5 (3.68)	.609
Depression (PHQ-8)	8.5 (5.23)	9.8 (5.14)	8.1 (5.20)	.023

a ATC = around-the-clock; BPI = Brief Pain Inventory; CAGE = Cut down, Annoyed, Guilty, Eye-opener; COMM = Current Opioid Misuse Measure; FACT-G = Functional Assessment of Cancer Therapy—General; LA = long-acting; MME = morphine milligram equivalent; NPQ = Neuropathic Pain Questionnaire; PHQ-8 = Patient Health Questionnaire-8; PRN = pro re nata, “as needed”; PSQI = Pittsburgh Sleep Quality Index Global Score.

### Participant-level pain flares trajectories

**Figure 1.**
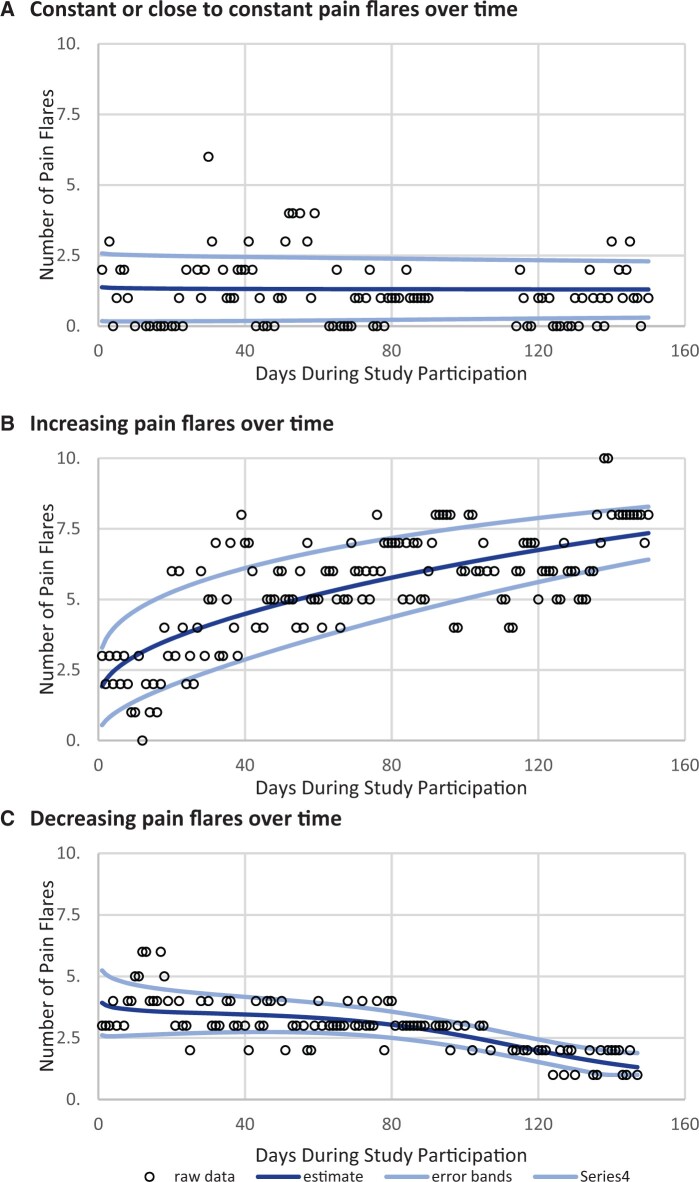
Examples of individual-level pain flare trajectories over study participation.

Participant-level trajectories fit 1 of the 6 categories: constant or close to constant, increasing, decreasing, concave, convex, and oscillating (Supplementary Table 1, available online). The majority of the participants (95%) demonstrated the 3 trajectories as shown in Figure 1.

#### Pain flare clusters

**Figure 2.**
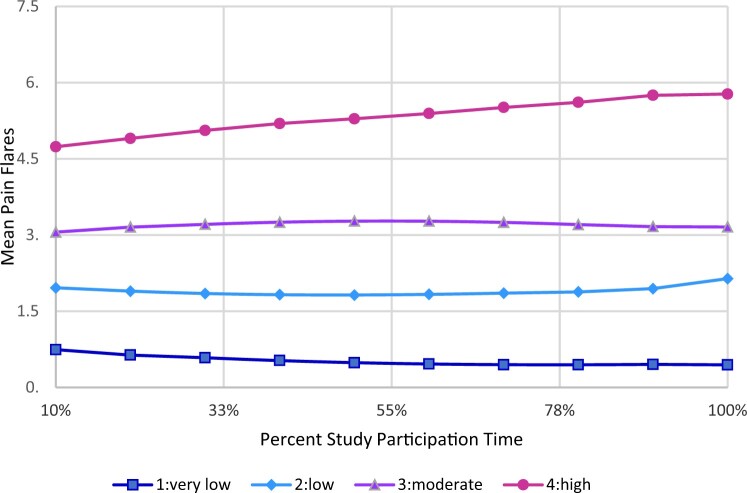
Plot of averages of pain flare means over proportional time during study participation for 4 identified clusters.

**Figure 3.**
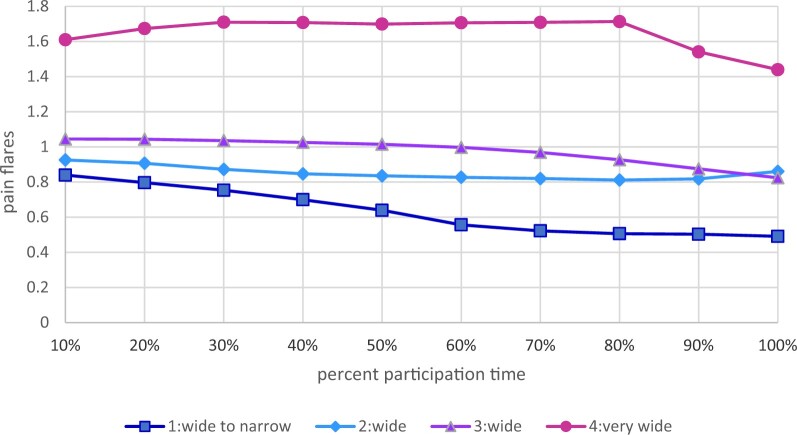
Plot of averages of pain flare half-widths over proportional time during study participation for 4 identified clusters.

Four pain flare clusters were identified. The mean distribution for these clusters and variability over time are reported in Figures 2 and 3, respectively. Combined, these plots indicate that the clusters represent increasing or more problematic levels of pain flares over time. Among the clusters with the highest mean pain flares, mean pain flares were greater than 4 across time (high-occurrence), whereas mean flares were less than 4 among the 3 other clusters (low-moderate occurrence) (Supplementary Table 2, available online).

### High-occurrence vs low-moderate occurrence clusters

Of the sample, 23% of participants were in high-occurrence pain clusters (cluster 4) having a mean of 5.5 (SD, 5.47) pain flares each day vs clusters 1-3 (77%), who had a mean of 2.4 (SD, 2.74) pain flares each day (*P* < .000). In total, 67.2% of patients receiving PRN-only opioids were in the “high-occurrence pain flare cluster” vs 27.9% receiving both PRN + ATC opioids (*P* = .024) (Table 1).

Patients in the high-occurrence pain flare cluster (compared with low-moderate occurrence) reported higher levels of worst pain (*P* = .000), least pain scores (*P* = .001), and greater pain-related interference (*P* = .000). Additionally, the Pain to Touch subscale of neuropathic pain was higher for those with high-occurrence flares compared with those with fewer flares (*P* < .000). Patients in the high-occurrence pain cluster also had poorer quality of life (*P* = .001) and lower self-efficacy in managing pain (*P* = .006). (Table 2).

### Predictors of high-occurrence pain flares

In the individual predictor models, several SDoH variables were significantly associated with high-occurrence pain flares, including income less than $30 000 compared to those earning more than $90 000, followed by being unemployed, being African American (compared with White or Caucasian), having a high school education or less, and having Medicaid insurance. Other significant variables included COMM score, baseline inpatient hospital stay duration, quality of life (FACT-G), and being prescribed a PRN-only opioid regimen (when compared with PRN + ATC regimens) (Table 3).

**Table 3.** Predictors of high pain flares cluster^a^

Predictor	Reference	OR	95% CI	*P*
Individually significant predictors				
African Americans	Whites/Caucasians	2.04	1.28-3.24	.001
High school or less	College, trade school, or more than college	2.17	1.39-3.39	.001
Income <$30 000	>$90 000	3.24	1.83-5.75	<.001
Unemployed, disabled, other	Employed	2.89	1.61-5.21	<.001
Medicaid insurance	Private Insurance	2.65	1.35-5.19	.005
Opioid misuse (COMM)		1.06	1.02-1.11	.006
Quality of life (FACT-G)		0.99	0.98-1.00	.041
Inpatient long stay (Yes)	No	2.02	1.13-3.61	.018
PRN only opioid prescription	Both PRN + ATC	1.59	1.10-2.51	.046
Multiple predictor model				
High school or less	College, trade school, or more than college	2.10	1.32-3.35	0.002
Unemployed, disabled, other	Employed or retired	2.31	1.45-3.70	0.001
COMM		1.05	1.01-1.10	0.022
Inpatient long stay (Yes)	No	2.06	1.13-3.77	0.019
PRN-only opioid prescription	Both PRN + ATC	2.02	1.28-3.20	0.003

a ATC = around-the-clock; COMM = Current Opioid Misuse Measure; FACT-G = Functional Assessment of Cancer Therapy–General; PRN = pro re nata, “as needed.”

R^2^ values for models of each individually significant predictor as a function of the other individually significant predictors (computed using logistic regression for dichotomous predictors and linear regression for continuous variables) ranged from 3.8% to 30.8%, indicating that multicollinearity was not a problem for the model on the basis of all these predictors. For the backward elimination adjusted multiple predictor model, patients with a high school education or less (odds ratio [OR] = 2.10, 95% confidence interval [CI] = 1.32 to 3.35; *P* = .002), unemployment (OR = 2.31, 95% CI = 1.45 to 3.70; *P* = .001), COMM score (OR = 1.05, 95% CI = 1.01 to 1.10; *P* = .022), an extended EMT inpatient duration (OR = 2.06, 95% CI = 1.13 to 3.77; *P* = .019), and PRN-only opioid regimen (OR = 2.02, 95% CI = 1.28 to 3.20; *P* = .003) remained significant predictors of high-occurrence pain flare cluster membership. The strength of PRN-only opioid regimen became stronger in the multiple predictor model controlling for other variables (Table 3).

## Discussion

This study is the first to characterize cancer pain flares over an extended observation period in ambulatory patients using a daily ecological survey approach. We identified unique pain flare clusters and reported on social, demographic, and clinical predictors of high-occurrence pain flare clusters offering insights that could potentially inform better clinical patient management approaches.

The findings underscore the crucial significance of various social determinants, such as an income below $30 000, unemployment, lower educational attainment, Medicaid insurance, and self-identification as African American, in explaining high-occurrence pain flares on an individual level. In the adjusted model, lower educational attainment and unemployment were significant SDoH factors predicting high-occurrence pain flares.

Both unemployment and lower educational attainment can significantly contribute to the exacerbation of pain flares. Educational attainment impacts employment disparities by shaping job opportunities, work conditions, income levels, and access to health insurance (50,51). Aside from financial strain, unemployment can disrupt daily routines, social connections, and limit access to pain medications, all of which can, in turn, worsen pain symptoms (20,52). Beyond financial strain and access, well-developed communication models to effectively convey complex medical information to individuals with diverse educational backgrounds are often missing, creating additional pain self-management challenges for patients (53).

Although not all social determinants retained their significance in the adjusted model, these variables continue to be intricately interwoven in our sociopolitical landscape (54,55). Overall, these findings point to a grave scenario of poor pain care for underserved patients with cancer, who already grapple with complex health and social challenges.

Aside from social factors, we found that the odds of experiencing higher pain flares were more than doubled for patients prescribed PRN-only opioids and that more than half of the patients in this study were prescribed PRN-only opioids even though oncology cancer pain guidelines recommend the use of ATC opioids in addition to immediate-release opioids at approximately 10% of the daily MME dose for BTP (56). Because of perceived confusion across cancer pain management guidelines (34,57), clinicians and oncologists might have genuine uncertainty about how best to manage cancer pain, especially using opioids. As the ASCO guidelines authors recently concluded, “Opioids have long been the foundation of cancer pain management, yet serious challenges to their use exist, including a striking lack of research to guide clinical practice in this population” (56).

As noted earlier, opioid prescription, especially prescription of long-acting opioids, has declined significantly in the United States, including among oncology practitioners (23-25,58). There are newer concerns that an increased number of cancer patients may be receiving insufficient treatment or remaining untreated for pain (25,58). For example, the majority of patients in our study reported moderate or severe pain intensity, especially those in the high pain flares cluster, but only 28% in the high-occurrence pain cluster were prescribed both ATC/PRN opioids. Attention to this subgroup is important, as there is accumulating evidence that poorly controlled pain increases the risk of suicide (59,60). This scenario is made worse by poor access to nonopioid, nonpharmacological treatments, such as complementary and integrative treatments for pain, especially by SDoH factors such as lack of insurance coverage and significant out-of-pocket costs (61,62).

Finally, our adjusted findings also point to a very small but significant role of opioid misuse factors in predicting high-occurrence pain flares. However, taken collectively (magnitude of pain in this sample and opioid prescription patterns), this finding might reflect the clinical phenomenon of poorly controlled baseline pain. In contrast to an opioid use disorder, opioid misuse may or may not be related to overuse of opioids (63). Indeed among patients with cancer, underuse of opioids, rather than overuse of opioids, has been found to be the main concern (20,64-66). Underuse of opioids in patients with cancer occurs due to opioid stigma, heightened fears of addiction, and lack of patient education on correct use of opioids (64,65). In fact, a significant number of cancer patients may have considerable gaps in opioid use, which may lead to both opioid safety risks and poor baseline pain control (64,65,67,68). Further studies are needed to understand how opioid prescription patterns may be associated with cancer pain control and trajectories of pain flares to generate an evidence base to inform clinical practice and cancer pain guidelines.

## Limitations

Investigating pain flare trajectories and predictors was an exploratory aim of our study, and our study sample was not powered for this purpose. Despite this, the findings point to pain flares as a significant clinical phenomenon with important actionable correlates. Because we collected pain flare data as part of daily ecological assessment, we limited the number of daily survey items to manage participant burden. Consequently, we did not collect detailed information on the types of pain flares or their onset, duration, and mitigating factors. Future studies may employ more comprehensive approaches to understanding pain flares, including patient perspectives, as well as their impact on more distal clinical outcomes, such as health-related quality of life and daily function. Although our study controlled for disease stage, our analysis of pain flares may not fully capture the complexity of interactions and variability in pain experiences across different cancer types and stages. We used the stage of disease as a proxy. However, this method may not fully adjust for nuanced differences in pain experiences specific to each type of cancer. These limitations should be considered when our findings are interpreted.

In this study, we examined the trajectories and clinical and SDoH factors influencing high-occurrence pain flares in ambulatory patients with cancer. Findings showed that PRN-only opioid regimens resulted in 102% higher odds of encountering pain flares compared with ATC opioids. Additionally, SDoH variables were predictors of higher pain flare occurrence. These insights are crucial in the current context where opioid prescriptions are declining and access to nonpharmacological treatments remains limited. The study underscores the pressing need for an evidence-based approach to managing pain flares that considers both prescription patterns and SDoH needs. This could better equip providers and services to mitigate acute pain intensification, including among vulnerable populations.

## Supplementary Material

pkae003_Supplementary_Data

## Data Availability

Deidentified data for the primary outcome will be made available via a request to the principal investigator with a data use agreement in accordance with the institutional policies.
