# Supplementary material for: Trajectories and predictors of high-occurrence pain flares in ambulatory cancer patients on opioids
Source: JNCI Cancer Spectr. 2024 Jan 24;8(1):pkae003. doi: 10.1093/jncics/pkae003 (PMC10880071; doi:10.1093/jncics/pkae003)

Supplementary Material

Supplementary Table 1. Distribution of Mean Pain Flares Trajectory Types

Supplementary Table 2. Distribution of Pain Flares Trajectory Clusters

Supplementary Figure 1. Study Strobe Chart

This supplemental material has been provided by the authors to give readers additional information about their work.

Supplementary Table 1. Distribution of Mean Pain Flares Trajectories

Mean trajectory type	Total (N = 270)
Constant or close to constant	137 (50.7%)
Increasing	68 (25.2%)
Decreasing	52 (19.3%)
Concave	8 (3.0%)
Convex	3 (1.1%)
Oscillating	2 (0.7%)

Supplementary Table 2. Distribution of Pain Flares Trajectory Clusters

**CLUSTERS**	**TOTAL (N = 270)**
Cluster 1 (Very Low)	88 (32.6%)
Cluster 2 (Low)	75 (27.8%)
Cluster 3 (Moderate)	45 (16.7%)
Cluster 4 (High)	62 (23.0%)

Supplementary Figure 1. Study Strobe Chart

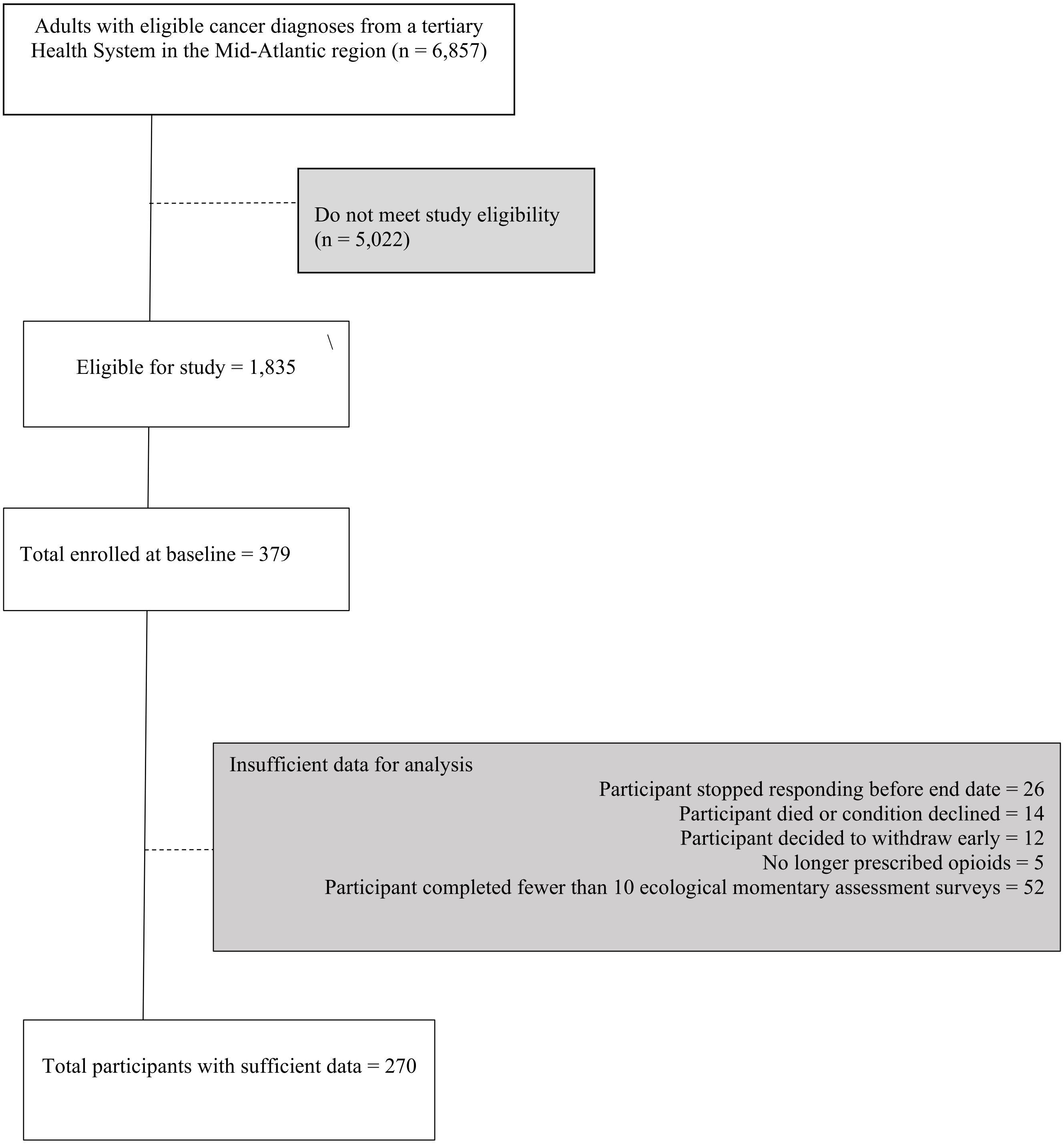

Supplement: pkae003_Supplementary_Data [file pkae003_supplementary_data.pdf]
